# Diagnostic and therapeutic wandering in the face of a pregnancy on cesarean scar

**DOI:** 10.11604/pamj.2024.47.173.43290

**Published:** 2024-04-09

**Authors:** Haithem Aloui, Rami Hammami

**Affiliations:** 1 Department 'C' of Gynecology and Obstetrics of Tunis Maternity and Neonatology Center, University of Tunis El Manar, Faculty of Medicine of Tunis, Tunis, Tunisia

**Keywords:** Pregnancy, cesarean scar, hemorrhage, conservative treatment

## Image in medicine

A 36-year-old patient with a history of cesarean section presented with a pregnancy halted at 8 weeks of gestation. Initial treatment with misoprostol (CYTOTEC*) failed to induce expulsion of the products of conception. Following a 24-hour observation period, a second misoprostol regimen also proved ineffective. Subsequently, ultrasound-guided aspiration was performed on day 4, revealing an isthmic gestational sac, an open cervix, and minimal dark bleeding. Initial aspiration yielded trophoblastic tissue, followed by profuse bright red hemorrhage. Intrauterine tamponade was achieved using a size 18 Foley catheter filled with 60 ml of saline and left in place for 48 hours. Hemoglobin levels dropped from 11 to 6 g/dl, necessitating transfusion with 4 units of packed red blood cells. After 14 days, the patient presented with hemorrhagic shock. Endovaginal ultrasound demonstrated significant abdominal hemorrhage and evidence of retained trophoblastic tissue. Intraoperatively, a pregnancy on a cesarean scar with uterine wall rupture was identified. Excision of the pregnancy and its myometrial bed was performed, followed by uterine reconstruction. The patient was discharged on postoperative day 2 with satisfactory clinical progress.

**Figure 1:**
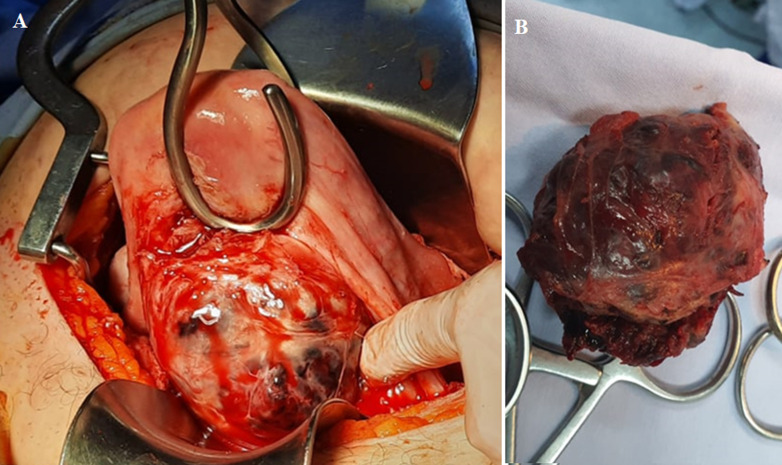
pregnancy on cesarean scar; A) intraoperatively; B) after excision

